# Modern Dentistry for the Laity

**Published:** 1921-02

**Authors:** Alfred A. Crocker


### Modern Dentistry for the Laity:

### Its Origin and Progress

By ALFRED A. CROCKER

I AM very glad that through my work on the subject of "Modern Dentistry for the Laity" I have attracted sufficient attention to be asked by the American Dental Dealers' Association to read a paper before them at this meeting on the subject.

The origin of the movement for the publicity on dental subjects rests upon a triangular base, very well grounded and of sound foundation. The three elementary parties to the propaganda are the public, the dental profession and the dental trade. Before systematic propaganda was started, the small fraction of the people having dental work done indicated great possibilities of benefits from better distribution of dental knowledge. The dentists in writing on the subject of care of the teeth and preventive dentistry frequently exclaimed, "Oh, if the people could only understand more about the importance of their teeth, how grand it would be!" This indicated that the dental profession were seeking some avenue of publicity for the necessity of their work but had not yet found it.

On account of the feeling in dental circles regarding advertising, the subject of ethical dental publicity remained an unsolved problem. The dental trade at that time seemed hedged off from the public without means of framing public opinion on the importance of their business, a privilege enjoyed by most every other line of trade you can think of.

The advertising by tooth preparation manufacturers has had the tendency to leave the impression on a great majority of the public that a tooth brush and a package of paste will preserve the teeth. In large type most pastes are heralded as tooth savers—and by association of ideas health preservers—and in small type the advertisements go on to say, "See your dentist semi-annually," which is indeed very kind to the profession. But as the real importance of the relative values of the tooth paste and dental service is just the other way, the dental service being the real tooth saver and health provider, it occurred to me that a public statement which would give all attention to the value of dental work and give the dentist all credit for the ability to preserve the teeth without but slightly mentioning tooth paste would be the fairer method. This led to my giving the dental profession large type representation and tooth pastes small type notice.

With the above as a state of the dormant forces the preparations for the war swept over the country, bringing every line of trade and profession to the front that were essential to the safety of the nation. Dentistry was named as essential, and many a recruit found out how essential it was when he received his notice of dental requirement and had to go to a dentist before he could be mustered in. This in itself was a complete demonstration of the possibilities of dental propaganda and pointed out to me one way it could be done. And the time had come when it could most easily be accomplished, for the word "preparedness" had assumed a larger meaning by including dentistry. It occurred to me that a dental supply man could take a hand in the propaganda of dentistry and do a great beneficial work to the country, could help the dental profession in explaining the subject of dentistry to the people, extoll the benefits derived from preventive dentistry, and explain the subject of focal infection —the same subject which had made the specialty professions of eye, ear, nose and throat so prominent—and at the same time bring about a thorough understanding of the value and importance of the dental business, the business which stands behind the dental profession and furnishes instruments, materials and supplies for the relief of humanity through their use in the hands of the dental profession. These fundamental ideas on which the whole subject is built and later took form in the first edition of my book, "Modern Dentistry for the Laity," were tested out as time went on and in the fall of 1917, by personal interviews with representatives of each of the three parties which form the foundation, I convinced myself that the idea was favorable to all parties concerned. During the spring of 1918 about fifteen articles were published and read by the public in magazines. Each article was a synopsis of the work performed by a dentist and explained the process of decay. Besides, the periodical visit to a dentist was explained as being not only for a tooth cleaning, but also to detect decay. Announcement of the progress of this work was made to the trade in June, 1918, when some of the articles which I had succeeded in having accepted for publication were reprinted in book form with a preface and index entitled, "Modern Dentistry for the Laity." This book was distributed to a list of incorporated industrial and mining companies and railroads, to all public libraries, to dentists and to the dental trade. From that time on the idea began to grow and the book was enlarged by additional papers that had been published by the time the second edition was printed. All lines of business eventually find a legitimate outlet for a statement of their essential place in the estimation of the public and standardize a method by which they can constantly maintain public opinion on the benefit of their offices to humanity.

The dental profession had for some years been carrying on children's clinics, which is a form of dental propaganda, for it reaches the home and directs the attention of the coming generation to the necessity of care of the teeth and the importance of seeing a dentist periodically. Though this was well taken care of by the dentists, it however left the adult population without direct appeal, and, as war work required the adults, the first subject to receive my attention was, "Dentistry in the Hospital." This subject was given an acceleration and impetus with articles in industrial magazines on the affiliated subject, "Industrial Dentistry," for many factories had medical service in small hospitals and were prospects for dental service as soon as the subject could be thoroughly understood. This was very timely and interesting to factories doing war work. "Industrial Dentistry" has been the means of reaching a great number of people with the gospel of dentistry in both the laboring and capital classes, and still forms one of the most effective methods of reaching large numbers of people. It is a business proposition and safety welfare work of the highest order, completing the hospital which is incomplete without it. A very large field besides this is the general hospitals, of which there are a great many, and of which very few have dental departments. Literature on this subject became necessary and led me to make several trips to hospitals, both with and without dental service. From the resulting interviews I wrote the first articles the Hospital Magazines ever printed on the subject, "Dentistry in the Hospital." Group diagnosis, the modern system in a modern hospital, requires opinions and findings by diagnosis of specialists in all branches of the healing art, and, of course, is incomplete without the dental clinician's report of tooth condition and his X-ray of the teeth, together with his recommendations. The recommendations of a hospital dental clinician cover certain first aid work he will do himself to clear the patient of the hospital and, besides, if necessary, he recommends further work for the patient's dentist to attend to when the patient is well again. With literature of this kind we have something with which we can reach the captains of industry and help them perfect their elaborate systems of safety welfare work, and with which we can reach the many hospitals not yet equipped for dental diagnosis or dental service. In carrying on this propaganda I have clung to the system of free distribution of the articles written for the general public, because the delivery of the subject is in the nature of a safety warning.

Any paper or magazine can get an article gratis for the asking. Besides, many pamphlets have also been distributed free. For those especially interested in this subject, the book contains all the information in condensed form, covering various phases of dentistry; preventive dentistry for the individual; see your dentist; industrial dentistry; dentistry in the hospital; focal infection; dentistry for life insurance and odontalysis, and meets the demand that has sprung up for this subject by those who seek it in the book stores and public libraries.

Knowing what we now know about the subject, I consider it a public trust to deliver the message to every one by every means possible. Instead of having humanity wait for the slow process of evolution of the unaided current thought to bring them to the subject of dentistry, dental propaganda is accomplishing the necessary work by direct and efficient methods, bringing the subject to them. At the present time the medical profession are giving great assistance to the subject by directing their patients to have a dental diagnosis. Several medical magazines have dental departments, edited by dentists. Cities have dental health department bulletins. The dental profession are very much interested in the movement and have contributed many articles for the press and magazines, and have addressed large audiences on the subject of care of the teeth and their relation to health. The American Dental Society formed a branch society for the industrial dental surgeons at the meeting, August, 1918. In August, 1919, the Industrial Dental Surgeons met with the National Safety Council. The Life Extension Institute of New York has added dental diagnosis to its medical supervision. Many of the factories using dental service print articles on the subject of care of the teeth in their service department weeklies.

When I took up this subject, only about forty corporations had established dental clinics for safety tooth work—now there are over one hundred and fifty. The National Safety Council has incorporated dental service and dental prophylaxis in their regular schedule for safety control. All this has taken place since 1917. Safety in the factory nowadays does not mean merely prevention from accidental injury, it means also prevention from injury that eventually comes to those not informed on various forces that are bound to affect their welfare if not pointed out by an overseeing superintendent or service director, who has at his command specialists in various professions and sciences making periodical examinations. Safety is the purpose of all welfare work and this now very properly includes preventive dentistry. The value of this work to the public is that it is educating and informing on a phase of public health, and points the way to prevention of tooth troubles. The value of this work to the dental profession is that it is an assistance to the work already being carried on in school clinics, and broadens the scope of dental publicity in a perfectly ethical way which the school clinic started. The value of this work to the dental trade is that it establishes with full sanction of both the public and dental profession a method by which the dental trade can assist the dental profession in the dissemination of the importance of seeing a dentist for care of the teeth. By the methods now started the various campaigns for teaching the laity about dentistry will surely make a wonderful change in the amount of dentistry performed, for annually, numerous people will be reached who otherwise would neglect that duty to themselves.

The subject of preventive dentistry is one that needs publicity to make it universal for preventive dentistry to prevent it will have to get to the people, and that only will come by publicity. This feature of the case makes it real missionary work and led to the expression of which I have made occasional use, that the purpose of this publicity being to tell something to the very people who should know it, places dental propaganda in the front rank of utilities for public service. Not only for health, but also for appearance, is dentistry a benefit to mankind. Dental methods of today make it possible for the grandparents to retain their youth and appear in public with just as pleasing appearance as their grandchildren. In dentistry the people find the fountain of youth. When it is realized that every person has teeth or wishes he had some, is not the subject of preventive dentistry interesting—the subject which tells how to save the teeth by seeing your dentist? A good tooth in the jaw is worth ten on a rubber plate, but on the other hand a set of false teeth is better than one tooth that is a focus of infection.

Educational work of this kind is a subject which grows as time goes on and will call for different methods, possibly to suit changing and progressing conditions, all of which can be taken care of when the time arrives. For the present the industrials and the hospitals are not by any means properly dentalized and present good fields to work in. World peace will give industries opportunity for closer study of dentistry and to plan their safety welfare along lines found so helpful during the war, by including dental as well as medical supervision and the hospitals, to meet the growing necessity of group diagnosis, will install dental equipment and retain dental clinicians. It is with the intention of helping this subject along that I have been carrying on the work of "Modern Dentistry for the Laity."

